# Quick Setup of Force-Controlled Industrial Gluing Tasks Using Learning From Demonstration

**DOI:** 10.3389/frobt.2021.767878

**Published:** 2021-11-05

**Authors:** Iñigo Iturrate, Aljaz Kramberger, Christoffer Sloth

**Affiliations:** SDU Robotics, Maersk McKinney Moller Institute, University of Southern Denmark, Odense, Denmark

**Keywords:** learning from demonstration, parameter estimation, force control, gluing, adaptive control

## Abstract

This paper presents a framework for programming in-contact tasks using learning by demonstration. The framework is demonstrated on an industrial gluing task, showing that a high quality robot behavior can be programmed using a single demonstration. A unified controller structure is proposed for the demonstration and execution of in-contact tasks that eases the transition from admittance controller for demonstration to parallel force/position control for the execution. The proposed controller is adapted according to the geometry of the task constraints, which is estimated online during the demonstration. In addition, the controller gains are adapted to the human behavior during demonstration to improve the quality of the demonstration. The considered gluing task requires the robot to alternate between free motion and in-contact motion; hence, an approach for minimizing contact forces during the switching between the two situations is presented. We evaluate our proposed system in a series of experiments, where we show that we are able to estimate the geometry of a curved surface, that our adaptive controller for demonstration allows users to achieve higher accuracy in a shorter demonstration duration when compared to an off-the-shelf controller for teaching implemented on a collaborative robot, and that our execution controller is able to reduce impact forces and apply a constant process force while adapting to the surface geometry.

## 1 Introduction

Learning from demonstration enables programming of a variety of robotic tasks in a way that is intuitive to a non-expert end-user. Previously, this method was utilized in an industrial context to teach robot based assembly tasks e.g., peg-in-hole insertion task, as well as polishing and grinding tasks. However, most existing approaches are applied on artificial or simplified benchmarks, and often do not consider the tight tolerances and process requirements with regards to the applied force that occur in a real manufacturing scenario. They often require multiple task demonstrations in order to learn task constraints or achieve robust task executions. Lastly, they often do not encompass the full pipeline of demonstration—encoding—execution from an end-user point of view.

In this paper we present a method for fast programming of a force-controlled industrial gluing task from a single user demonstration. We propose the use of adaptive admittance control during the demonstration phase to enable the user to kinesthetically move the robot more intuitively by providing a better control response, allowing them to provide more accurate task demonstrations.

From a user demonstration of the task, we obtain information on the kinematic trajectory of the robot. This allows us to then encode the motion in a motion primitive that allows both direct replay and generalization of the task. By detecting the contact state of the robot and estimating the surface normal, we can segment the task into phases of unconstrained (free-air) and constrained (in-contact) movement. Simultaneously, we estimate the constrained axes of the robot along the trajectory. This allows us to formulate a parallel position/force controller for the subsequent execution of the task, such that we can either imitate the forces applied during the demonstration or allow the user to specify a constant contact force, as is typical in industrial applications.

**FIGURE 1:**
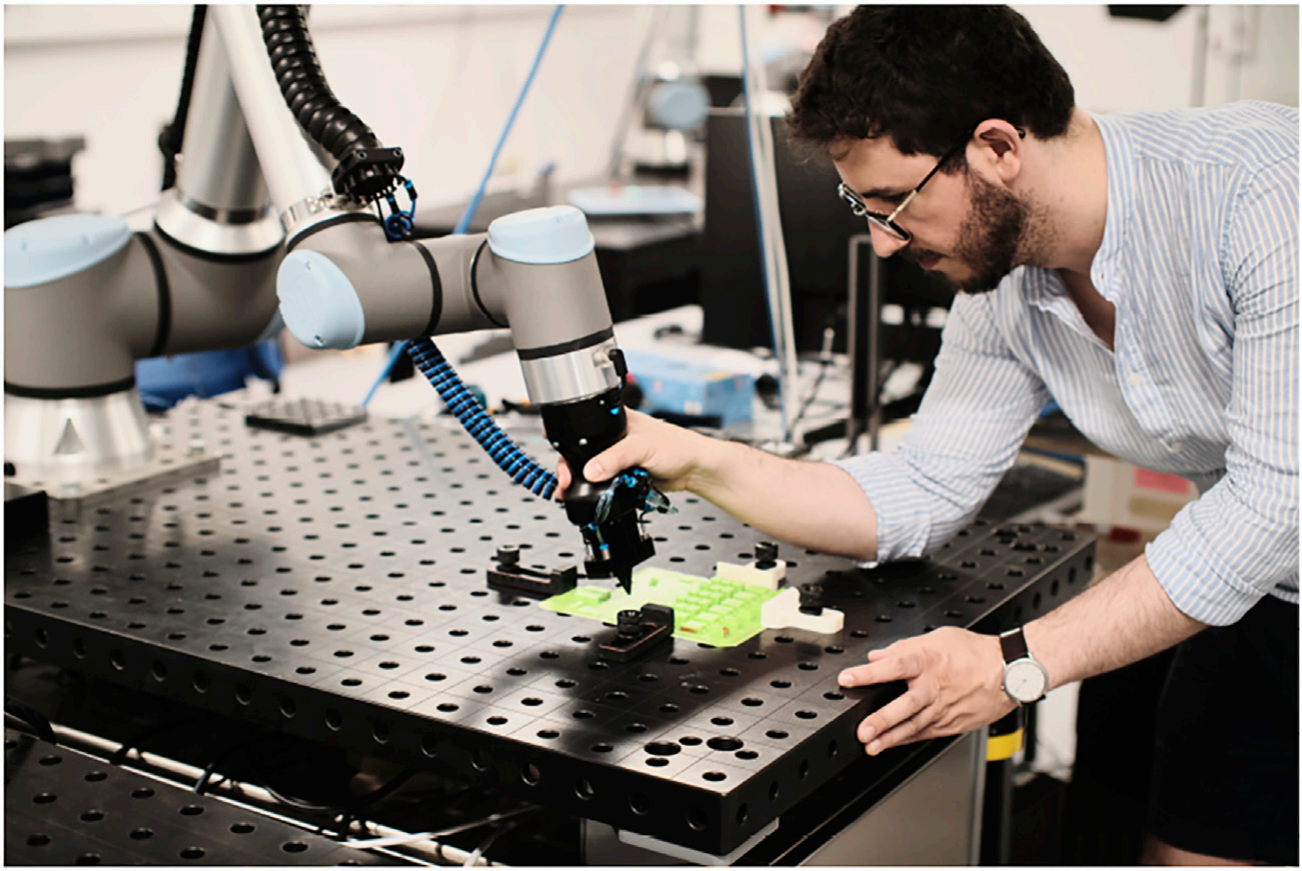
User demonstration of a gluing-like task on our benchmark system.

We evaluate our system on a benchmark that is closely based on a real industrial gluing task related to printed circuit board assembly, as shown in Figure 1, showing that our method can ensure constant and safe interaction forces and suitable trajectories in a task with low contact-force requirements.

## 2 Related Work

With the evolution of collaborative robots, new user-friendly efficient and flexible ways for automating industrial tasks which were previously done with specialized automation equipment are possible (Gašpar et al., 2020). Furthermore, methods such as Learning by demonstration (LbD) (Billard et al., 2008) and force control can be exploited, thanks to the integrated sensors and controllers of the collaborative robot systems.

In robotic learning by demonstration scenarios, robot trajectories that describe the kinematic information are recorded (Deniša and Ude, 2015). On the other hand, for tasks associated with industrial robotics e.g., assembly (Abu-Dakka et al., 2015) or polishing (Gams et al., 2013), dynamic data is equally important. These two data sets lay the basis for in-contact execution of robot tasks, where the learned trajectory cannot be replayed but has to be adaptable to changes in the environment. To facilitate this requirement, in a lot of works, robot trajectories are represented with Dynamic Movement Primitives (Ijspeert et al., 2013; Ude et al., 2014). With this framework, both kinematic and dynamic trajectories can be represented in a unified manner, and can be enhanced with additional properties such as modulation and time scaling.

The benefits of the DMP framework can be efficiently exploited for in-contact tasks, when coupling the DMP in a force-based LbD scenario, as shown in Koropouli et al. (2012) and Kormushev et al. (2011). In the work of Rozo et al. (2013) a two stage force LbD approach was presented, where in the first stage they recorded the positions and orientations of the desired movement and in the subsequent stage the corresponding forces and torques.

Simultaneous control of force and position for teaching and execution tasks is physically impossible (Stramigioli, 2001), and therefore these approaches all have to make compromises between prioritising force control or position control. In literature, classical position/force controllers are still adopted for dealing with this challenge. One of the early works in this field was presented by Asada and Izumi (1989). The measured data was used to automatically program the position and force set points for the hybrid force control framework (Raibert and Craig, 1981) used in Cartesian space. Furthermore, simultaneous position and force control frameworks in the LbD literature primarily focus on selecting the dimension of the constraint frame on which the force or position control is applied (Peternel et al., 2017) and the selection of the best control frame (Ureche et al., 2015).

The main motivation for constraint selection methods is that the dimension of the constraint frame that is exposed to a high variance in force and low variance in position should favor force control, and vice versa (Ureche et al., 2015). Constraint frames are usually chosen manually based on the task specifications Raibert and Craig (1981). Common choices include the tool frame (Kronander and Billard, 2014; Peternel et al., 2017) and the surface normals (Deng et al., 2016; Conkey and Hermans, 2019). The latter can also be estimated, e.g. from the velocity of the contact point (Karayiannidis et al., 2014; Sloth and Iturrate, 2021). Conkey and Hermans (2019) present a framework for learning a dynamic constraint frame aligned to the direction of desired force using CDMPs and force control to ensure that the force is aligned in the normal plane with the direction of the movement. In (Kober et al., 2015), multiple demonstrations of a task are used to segment a series of separate movement primitives, each associated with a specific discrete frame of reference. The transitions between each of the primitives—and therefore compliant frames—are then separately addressed. Kumar and Rani (2021) present a new hybrid force/position control approach for time-varying constrained re-configurable manipulators for physical human robot interaction with environment of unknown stiffness. A review of hybrid position/force controllers is presented in Ortenzi et al. (2017).

An alternative to hybrid force/position control is impedance control with varying parameters. By changing the stiffness parameters of the impedance controller, the interaction forces can be adjusted between the robot and the environment (Buchli et al., 2011). Stiffness adaptation can be learned from demonstration in multiple ways, and typically requires multiple demonstrations or iterative corrections of the path. In Lee et al. (2015), the mean and covariance information of the trajectories from multiple demonstrations of a task are used as an approximation of the desired stiffness along different regions of the path. Kronander and Billard (2012, 2014) allow the user to iteratively re-execute the task and apply external disturbances on the robot such that it deviates from the demonstrated path. The magnitude of these deviations is then used analogously to stiffness. Pastor et al. (2011) introduced a method for real-time adaptation of demonstrated trajectories for task exertions, depending on the measured sensory data. They developed an adaptive regulator for learning and adaptation of demonstrated motion, where actual and learned force feedback was utilized. Schindlbeck and Haddadin (2015) presented a unified impedance approach for safe online parameter adaptation, based on passivity and energy tanks. Furthermore, a review of online adaptation of impedance control parameters for human robot interaction is presented in (Ficuciello et al., 2015; Müller et al., 2018) and in-contact execution tasks are presented by Abu-Dakka and Saveriano (2020). To adapt the controller parameters one can estimate the compliance of the interaction between robot and environment. This approach is taken in Santos and Cortesão (2018), where the perceived stiffness is estimated and used for controller gain scheduling.

For tasks where impedance control cannot be utilized because of the physical limitation of the used systems, admittance control (Siciliano et al., 2009) can be used for teaching and execution of tasks in contact with the environment. A variable admittance controller, for safe human-robot interaction, where the admittance control parameters were adapted based on the passivity criterion was presented by Ferraguti et al. (2019). Furthermore, variable admittance control can be adopted in human-robot cooperation tasks by means of learning (Dimeas and Aspragathos, 2014, 2015). Finally, Li et al. (2020) consider the robot to be in contact with both human and environment, similar to the condition considered in this paper.

It is important to ensure the stability and guarantee satisfactory performance despite varying controller gains and environmental compliance. Elegant stability conditions are set up in Müller et al. (2019) for a physical human-robot system with delays in the human reaction. The system considered in this paper has varying parameters in addition to input delays; thus, the methods presented in Briat (2014) may be used for the stability analysis and performance specification.

Recently, automated gluing applications have become very popular with the development of new industrial machines and tools, which can be efficiently utilized by collaborative robots. The majority of the work focuses on defining the robot trajectories based on the CAD description of the industrial object (Castelli et al., 2021). Other approaches, utilize simple Cartesian space industrial manipulators coupled with computer vision (Pagano et al., 2020). The vision system enables localization and reconstruction of the shape of objects to which glue should be applied. The gathered information is used to construct a motion trajectory that can be executed with the specialized manipulators, achieving the desired industrial performance.

## 3 Problem Formulation and Contributions

Given a gluing or similar in-contact processing or dispensing task, our objective is to develop a full pipeline comprising the demonstration, encoding and execution that enables a robust and high-quality execution of the task with as little setup as possible required from the end-user. In order to make the approach more universally deployable in an industrial setting, we propose to learn from a single user demonstration, while exploiting the semantics of the task to adapt the robot controllers.

A key characteristic of the addressed task is that it is composed of free-air approach and retraction motions, but the actual dispensing or gluing consists in an in-contact motion whereby the manipulator is partially constrained by the environment. In particular, this constraint will be given by a surface normal during contact. In addition to this, in the proposed learning from demonstration scenario using kinesthetic teaching, the human is also in contact with the robot, thus partially constraining its motion as well.

**FIGURE 2:**
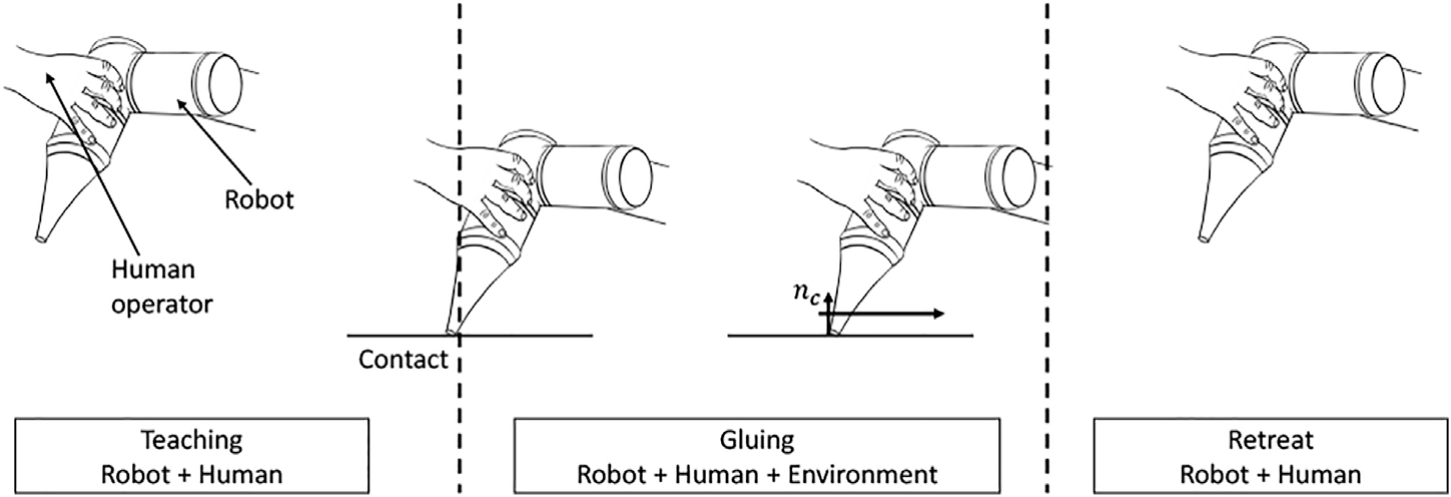
Idea of the approach.

An illustration of how these concepts fit into our system pipeline is shown in Figure 2. From an initial approach motion consisting only of the robot in contact with the human, the human will then move the robot into contact with the target surface and perform a sliding motion for the actual dispensing. This will correspond to a state where the entire system dynamics are comprised of the robot, the human and the environment. Finally, a retraction motion will move the robot away from the surface and into a final state where the dynamics will be given by the robot and human. If we consider the subsequent autonomous execution of the task, the dynamics will be the same, with the exception of the human’s contribution.

We consider our main contribution to be our controller design for kinesthetic teaching of processing tasks. We propose an adaptive controller architecture that takes into account the different constraints of the task at different phases of the execution. In particular, our approach to demonstration comprises the following:

1) We learn continuous kinematic and dynamic task constraints from a single demonstration.
2) We use variable-gain admittance control for kinesthetic teaching, where the damping is continuously adapted based on a velocity-force rule in order to match the user’s intention and reduce the physical effort needed during teaching.
3) We couple this controller with a contact detection algorithm, such that we are able to detect the start of the dispensing phase and increase controller damping to guarantee stability, even in cases of sudden impact with a stiff environment.
4) Once contact with a surface is established, we additionally couple our gain scheduler with a surface normal estimator. The formulation of this estimator is guaranteed to converge given adequate excitation and furthermore guarantees the preservation of the unit norm. By estimating the surface normal, we are able to adapt the eigenstructure of gain matrices, such that we maintain higher damping in the direction(s) constrained by the environment, while being able to lower the damping—and thus assist the user—in the non-constrained directions.

In addition to our controller for demonstration, we also propose a second controller for task execution. This controller adopts a similar structure to the one used for teaching, albeit with a few modifications, and exhibits the following characteristics:

1) We use parallel position/force control to guarantee that both the kinematic and dynamic targets of the task are met.
2) Task kinematics are encoded as Dynamic Movement Primitives (DMPs). This allows us to learn from a single demonstration, while adopting an encoding that enables modulation of the motions. We specifically use modulation of the DMP goal to ensure a smooth transition between the approach and process motions, and to reduce the impact forces upon first contact with the target surface.
3) Task dynamics are preserved by encoding the output of the normal estimator during demonstration as Radial Basis Functions (RBFs) synchronized with the DMPs. By applying a user-defined process force magnitude in the direction of the encoded normal vector, we ensure that contact with the surface is maintained. We also use this information to vary the gains of the parallel position/force controller, such that force control is applied only on the normal axis, while the other axes are position-controlled.

### 3.1 Comparison to Other Approaches

Our work is conceptually similar to Kronander and Billard (2012); Kober et al. (2015); Lee et al. (2015); Conkey and Hermans (2019), in that we are interested in learning task constraints from demonstration, such that we can then adapt the parameters/gains of a compliant controller to the structure of the task. However, in contrast to other approaches (Kronander and Billard, 2012; Kober et al., 2015; Lee et al., 2015), which require either multiple (typically more than three) demonstrations of the task or multiple execution iterations in order to determine these constraints, our approach can do so from a single demonstration by exploiting the surface normal estimator to determine the task geometry. We consider this one of the main contributions of our proposed approach, as single-demonstration LbD methods are more widely applicable in industrial scenarios.

Our work is methodologically most similar to Conkey and Hermans (2019). While their method is also able to learn task constraints from a single demonstration, they rely on the forces observed during the demonstration to do so. Such an approach assumes that the demonstration forces correspond with the forces of an ideal execution and, furthermore, does not consider that the forces measured by the sensor during demonstration will not only be task/environmental forces, but will also be coupled with those applied by the human demonstrator to kinesthetically move/teach the robot. These two sets of forces will not always be aligned; thus, using them to learn task constraints will result in an alignment error in the constraint frame. By using the estimated surface normal as the desired direction that the forces should be applied in, our approach does not suffer from this coupling. The magnitude of the desired force can simply be specified by the user or could, e.g., be computed from the average of the magnitude of the demonstration forces in the normal direction.

As indicated above, in order to reduce contact force transients during the execution, we modulate the goal of the DMP slowly into the contact surface. Although this method is similar to Conkey and Hermans (2019), our use of it is qualitatively different. Whereas Conkey and Hermans (2019) use goal modulation reactively to adapt to unforseen generalizations of the task—i.e., a contact that is established either earlier or later than anticipated—we do so proactively by purposely setting the initial goal higher than originally demonstrated, to ensure that impact forces are always reduced.

Note also that contrary to the above-mentioned works by using surface normal estimation as the basis for determining task constraints, our approach is able to use these constraints not only to adapt the controller during the execution but also during the demonstration phase to actively aid the user. This is, indeed, not possible for approaches that determine task constraints a posteriori based on positional variance or segmentation (Kronander and Billard, 2012; Kober et al., 2015; Lee et al., 2015).

## 4 Methodology

The purpose of this section is to describe the proposed approach to obtain high-quality robotic gluing based on learned behaviors from kinesthetic teaching. It is challenging to learn the gluing behavior, as it is a task that requires contact between robot and environment; thus, both the motion and environment geometry and dynamic parameters should be learned from a single demonstration. As it is chosen to use only one demonstration for learning the task, the parameters estimated from the demonstration should be improved from executions of the task. We describe the entire process of 1) Programming by demonstration, 2) Encoding of robot behavior, and 3) Execution of desired gluing control; however, the main focus of the paper is the demonstration phase, as this is the novel contribution of the paper.

**FIGURE 3:**
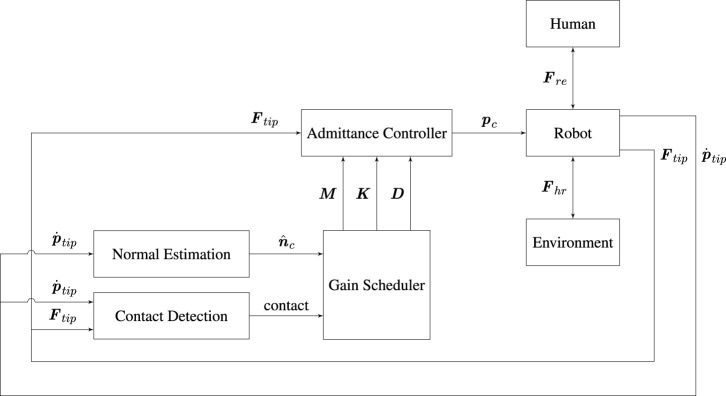
Block diagram of the controller architecture used for task demonstration.

We propose a unified controller structure for the kinesthetic teaching and execution of tasks with interaction between robot and environment. Figure 3 is a block diagram of the controller that runs during demonstration. The controller is an adaptive admittance controller, where the adaptation is conducted based on the surface normal to the environment when the robot and environment is in contact. This ensures stability and improves the quality of the demonstrated behaviors. A similar control structure is used for the execution, as shown in Figure 5; however, a parallel position/force controller is used instead of the admittance controller.

### 4.1 Adaptive Admittance Control for Kinesthetic Demonstration

The purpose of this section is to describe the admittance controller that is used for the demonstration of the gluing task. The admittance controller uses the notation from Caccavale et al. (1999) and is shown below

$$\left[\begin{array}{cc} M_{p} & 0\\ 0 & M_{o} \end{array}\right]\left[\begin{array}{c} \Delta {\ddot{p}}_{cd}\\ \Delta {\dot{\omega }}_{cd}^{d} \end{array}\right]+\left[\begin{array}{cc} D_{p} & 0\\ 0 & D_{o} \end{array}\right]\left[\begin{array}{c} \Delta {\dot{p}}_{cd}\\ \Delta \omega_{cd}^{d} \end{array}\right]+\left[\begin{array}{cc} K_{p} & 0\\ 0 & K_{o} \end{array}\right]\left[\begin{array}{c} \Delta p_{cd}\\ \epsilon_{cd}^{d} \end{array}\right]=\left[\begin{array}{c} f\\ \mu^{d} \end{array}\right],$$ (1)

where *M*_*p*_, *M*_*o*_, *D*_*p*_, *D*_*o*_, *K*_*p*_, *K*_*o*_ are 3 × 3 matrices, $f\in R^{3}$ is the force applied to the end-effector of the robot given in Base frame, Δ*p*_*cd*_ = *p*_*c*_ − *p*_*d*_, $\mu^{d}\in R^{3}$ is the torque applied to the end-effector given in desired frame, Δ*ω*_*cd*_ = *ω*_*c*_ − *ω*_*d*_, and $\epsilon_{cd}^{d}=\eta_{d}\epsilon_{c}-\eta_{c}\epsilon_{d}-S\left(\epsilon_{c}\right)\epsilon_{d}$ . The skew-symmetric operator is denoted by *S* and the unit quaternion is (*η*, *ϵ*), where *ϵ* is the vector-part.

The objective of this paper is to adapt the controller according to the interaction with both human and environment. In particular the interaction with the environment can cause instability if the controller gains are not adapted. Therefore, the surface normal is estimated during demonstration to allow the damping of the controller to be increased only in the direction of the environment.

#### 4.1.1 Surface Normal Estimation

The surface normal is estimated during contact using the following sliding condition

$$n_{c}^{T}{\dot{p}}_{c}=0,$$ (2)

where *n*_*c*_ is the surface normal and ${\dot{p}}_{c}$ is the velocity of the contact point given by

$${\dot{p}}_{c}={\dot{p}}_{e}+S\left(\omega \right){R_{e}}^{e}r,$$ (3)

and *ω* is the angular velocity of the end-effector, and *R*_*e*_ is a rotation matrix from base frame to end-effector frame, and ^*e*^ *r* is a vector from the end-effector to the contact point, which is assumed to be constant.

Since ^*e*^ *r* is assumed to be known, the estimate of *n*_*c*_ can be accomplished based on the estimation method presented in Karayiannidis et al. (2014) summarized in the following proposition. The proposition uses the projection matrix onto the orthogonal complement of a column vector $a\in R^{3}$ , defined as $\bar{P}:a\mapsto I-aa^{T}$ , where *I* is a 3 × 3 identity matrix.

Proposition 1 *The integral adaptive law*

$$\begin{array}{ll} {\dot{\hat{n}}}_{c} & =-\gamma_{n}\bar{P}\left({\hat{n}}_{c}\right)L_{n}\left(t\right){\hat{n}}_{c} \end{array}$$ (4)

$$\begin{array}{ll} {\dot{L}}_{n} & =-\beta_{n}L_{n}+\frac{1}{1+\|{\dot{p}}_{c}{\|}^{2}}{\dot{p}}_{c}{\dot{p}}_{c}^{T},\;L_{n}\left(0\right)=0, \end{array}$$ (5)

*where* $L_{n}\in R^{3\times 3}$ *guarantees that*

1) *the norm of the estimate* ${\hat{n}}_{c}\left(t\right)$ *is invariant, i.e., given that* $\left\|{\hat{n}}_{c}\left(0\right)=1\right\|$ *,* $\|{\hat{n}}_{c}\left(t\right)\|=1$ *for all* *t* ≥ 0
2) *if* $\vartheta \left(0\right)\in \left(-\frac{\pi }{2},\frac{\pi }{2}\right)$ *then* $\vartheta \left(t\right)\in \left(-\frac{\pi }{2},\frac{\pi }{2}\right)$ *for all* *t* > 0*, where* *ϑ* *is the angle between* *n*_*c*_ *and* ${\hat{n}}_{c}$

3) $\lim_{t\to \infty }\|{\dot{\hat{n}}}_{c}\|=0$

4) *if* ${\dot{p}}_{c}$ *is persistently excited then* *ϑ* *converges to zero exponentially which implies that* ${\hat{n}}_{c}$ *converges exponentially to* *n*_*c*_ *with a rate that can be tuned by* *γ*_*n*_ *.*

The proposition is based on Theorem 4.3.3 in Karayiannidis et al. (2014) and ensures that ${\hat{n}}_{c}$ remains a unit vector. The surface normal estimate is initialized based on the velocity ${\dot{p}}_{c}$ when contact is detected, i.e.,

$${\hat{n}}_{c}\left(0\right)=\frac{{\dot{p}}_{c}\left(0\right)}{\left\|{\dot{p}}_{c}\left(0\right)\right\|}.$$

The projection matrix $\bar{P}\left({\hat{n}}_{c}\right)$ in Eq. 4 ensures that the norm of ${\hat{n}}_{c}$ is preserved, since ${\dot{\hat{n}}}_{c}$ is tangent to the unit sphere; thus, we can compute the instantaneous angular velocity *ω*(*t*) from ${\dot{\hat{n}}}_{c}\left(t\right)$ and ${\hat{n}}_{c}\left(t\right)$ as

$$\omega \left(t\right)={\hat{n}}_{c}\left(t\right)\times {\dot{\hat{n}}}_{c}\left(t\right)$$

This property must be preserved when implementing the algorithm in discrete time. To accomplish this, we exploit the instantaneous angular velocity *ω*(*t*) for the update of the surface normal estimate. In particular, we implement the surface normal estimation using the following difference equations:

$$\begin{array}{ll} {\hat{n}}_{c}\left(k+1\right) & =e^{T_{s}S\left(\omega \left(k\right)\right)}{\hat{n}}_{c}\left(k\right) \end{array}$$ (6)

$$\begin{array}{ll} L_{n}\left(k+1\right) & =\left(1-T_{s}\beta_{n}\right)L_{n}\left(k\right)+T_{s}\frac{1}{1+\|{\dot{p}}_{c}{\|}^{2}}{\dot{p}}_{c}{\dot{p}}_{c}^{T},\;L_{n}\left(0\right)=0 \end{array}$$ (7)

where *T*_*s*_ is the sample time and

$$\omega \left(k\right)={\hat{n}}_{c}\left(k\right)\times \left(-\gamma_{n}L_{n}\left(k\right){\hat{n}}_{c}\left(k\right)\right).$$

#### 4.1.2 Contact Detection

A statistical method is used for contact detection; in particular, we consider the signal

$$\varphi ={\dot{p}}_{\text{tip}}^{T}f_{\text{tip}},$$ (8)

which will be negative when a collision occurs since a force is applied in the opposite direction of the motion. To achieve a robust collision detection, the non-restarting cumulative sum method (CUSUM) is applied to *φ* (Gandy and Lau, 2013).

#### 4.1.3 Parameter Adaptation

This section describes how the gains of the admittance controller are changed based on the dynamics of the demonstrator and the environment. Our approach is to change the eigenaxis of the parameter matrices according to the surface normal, and change the eigenvalues according to a force-dependent heuristic.

There exist multiple approaches for adapting the gains of an admittance controller, including force-dependent variable impedance control (Müller et al., 2018), velocity-dependent variable impedance control (Ficuciello et al., 2015), and passivity-based approaches (Ferraguti et al., 2019).

A force-dependent variable impedance control makes it easier to accelerate and decelerate the robot, as the damping is given by (Müller et al., 2018):

$$D_{VIC}\left(\Gamma \right)=-\alpha diag\left(\Gamma \right)diag\left(\mathrm{sign}\left(\dot{x}\right)\right)+D_{0},$$ (9)

where Γ is the wrench vector, *α* > 0 is a tuning parameter, $\dot{x}$ is the Cartesian velocity, and *D*_0_ is a diagonal matrix.

To ensure stability of the impedance controller when interacting with a human that varies the active damping and stiffness, a lower bound can be found for the damping that ensures stability; this is denoted *D*_*obs*_. Finally, the damping of the force-dependent variable impedance control is given by

$$D=\max\left\{D_{VIC},D_{obs}\right\}.$$ (10)

To analyze the stability of the impedance controlled robot during interaction with both environment and human, one can describe the system as a linear parameter varying (LPV) system. The varying parameters include the damping of the impedance control, the compliance of the human and the compliance of the environment. The stability of such systems has been considered in several work, including Müller et al. (2019), but here several parameters are considered to be constant. The presented stability criterion relaxes this assumption.

The model used in similar physical human-robot collaboration works such as Müller et al. (2019) is linear, but most of the gains are varying in the considered application. In particular, the compliance parameters of the human and stiffness of the environment may vary. These parameters may be estimated or considered to be unknown but bounded to some set of feasible parameter values. Also the damping of the controller is varying but known (in the force-dependent variable impedance control, it depends on the measured wrench). Finally, delays are present in the system originating from the active response of the human and delays in the control software.

The variations in the system dynamics may be modelled using an *N*-dimensional exogenous variable *ρ*(*t*), but since the response of the controller may be delayed compared to the response of the system, the delayed variable *ρ*_*h*_(*t*) = *ρ*(*t* − *h*) is introduced where $h\in \left[0,\bar{h}\right]$ is a constant time-delay.

Due to the linearity of the considered system dynamics, the impedance controlled robot can be modelled as the following LPV system with time-delays, see Briat (2014),

$$\begin{array}{ll} \dot{x}\left(t\right) & =A\left(\rho \left(t\right),\rho_{h}\left(t\right)\right)x\left(t\right)+A_{h}\left(\rho \left(t\right),\rho_{h}\left(t\right)\right)x\left(t-h\right)+E\left(\rho \left(t\right),\rho_{h}\left(t\right)\right)w\left(t\right) \end{array}$$ (11)

$$\begin{array}{ll} z\left(t\right) & =C\left(\rho \left(t\right),\rho_{h}\left(t\right)\right)x\left(t\right)+C_{h}\left(\rho \left(t\right),\rho_{h}\left(t\right)\right)x\left(t-h\right)+F\left(\rho \left(t\right),\rho_{h}\left(t\right)\right)w\left(t\right) \end{array}$$ (12)

$$\begin{array}{ll} x\left(s\right) & =\phi \left(s\right),\;s\in \left[-\bar{h},0\right], \end{array}$$ (13)

where *x* is the state of the system, *w* is a disturbance input, *z* is the performance output, *A*, *A*_*h*_, and *E* define the system dynamics, and *C*, *C*_*h*_, and *F* define the performance measure, and *ϕ*(*s*) provides the initial condition. The value of the parameter vector *ρ* must be within the compact set Δ_*ρ*_, and the rate of variation of the parameters should be within the set Δ_*v*_ = [−1,1]^*N*^. To simplify notation, we define the Hermitian operator (for real matrices) as $He\left(A\right)=A+A^{T}$ , denote the set of positive definite *n* × *n* matrices by $S_{≻0}^{n}$ , and define the following sets:

$$\begin{array}{ll} P^{v} & =\left\{\rho :R_{+}\to \Delta_{\rho }:\;\dot{\rho }\left(t\right)\in \Delta_{v},\;t\geq 0\right\} \end{array}$$ (14)

$$\begin{array}{ll} P_{h} & =\left\{\left(\rho ,\rho_{h}\right):R_{+}\to \Delta_{\rho }\times \Delta_{\rho }:\;\rho \in P^{v},\rho_{h}\left(t\right)=\rho \left(t-h\right),t\geq 0\right\} \end{array}$$ (15)

$$\begin{array}{ll} \Delta_{\rho }^{h} & =\left\{\left(\rho ,\rho_{h}\right)\in \Delta_{\rho }\times \Delta_{\rho }:\;|\rho_{hi}-\rho_{i}|\leq \bar{h},\;i=1,\ldots ,N\right\}. \end{array}$$ (16)

The stability of the time-delayed LPV system can be analyzed by the use of the following theorem (Briat, 2014, Theorem 6.3.3), which is based on identifying a parameter-dependent Lyapunov-Krasovskii functional due to the existence of time-delays. Also, a desired performance can be specified via a mixed sensitivity description and optimized by minimizing *γ* in the following theorem.

Theorem 1 *Assume that there exists a continuously differentiable matrix function* $P:\Delta_{\rho }^{h}\to S_{≻0}^{n}$ *, a matrix function* $Q:\Delta_{\rho }\to S_{≻0}^{n}$ *, a constant matrix* $R\in S_{≻0}^{n}$ *and a scalar* *γ* > 0 *such that the LMI*

$$\left[\begin{array}{ccccc} \psi_{11} & \psi_{12} & P\left(\rho ,\rho_{h}\right)E\left(\rho ,\rho_{h}\right) & C{\left(\rho ,\rho_{h}\right)}^{T} & \bar{h}A{\left(\rho ,\rho_{h}\right)}^{T}R\\ {}* & -Q\left(\rho_{h}\right)-R & 0 & C_{h}{\left(\rho ,\rho_{h}\right)}^{T} & \bar{h}A_{h}{\left(\rho ,\rho_{h}\right)}^{T}R\\ {}* & * & -\gamma I_{p} & F\left(\rho ,\rho_{h}\right) & \bar{h}E{\left(\rho ,\rho_{h}\right)}^{T}R\\ {}* & * & * & -\gamma I_{q} & 0\\ {}* & * & * & * & -R \end{array}\right]≺0$$ (17)

*holds for all* $\rho ,\rho_{h}\in \Delta_{\rho }^{h}$ *and all* *ν*, *ν*_*h*_ ∈ {−1,1}^*N*^ ×{−1,1}^*N*^ *where*

$$\begin{array}{ll} \psi_{11} & =He\left[P\left(\rho ,\rho_{h}\right)A\left(\rho ,\rho_{h}\right)\right]+\overset{N}{\underset{i=1}{\sum }}\left(\nu_{i}\frac{\partial P\left(\rho ,\rho_{h}\right)}{\partial \rho_{i}}+\nu_{hi}\frac{\partial P\left(\rho ,\rho_{h}\right)}{\partial \rho_{hi}}\right)+Q\left(\rho \right)-R \end{array}$$ (18)

$$\begin{array}{ll} \psi_{12} & =P\left(\rho ,\rho_{h}\right)A\left(\rho ,\rho_{h}\right)+R \end{array}$$ (19)

*Then the LPV system is asymptotically stable for all constant delays* $h\in \left[0,\bar{h}\right]$ *and* $\left(\rho ,\rho_{h}\right)\in P_{h}$ *and the* *L*_2_ *-gain from* *w* *to* *z* *is less than* *γ* *.*

Theorem 1 applies to the considered robotic system; however, to ease the computation of the stability condition, the system model should be written as a polytopic LPV system. Then it is sufficient to evaluate Eq. 17 at the vertices of the parameter space, see Briat (2014) for details.

### 4.2 Learning Task Kinematics

In order to encode a gluing trajectory from a user demonstration in a flexible manner that will allow us to reparameterize and reuse the learned task, we adopt Dynamic Movement Primitives as our representation for robot kinematic trajectories.

#### 4.2.1 Cartesian-Space Dynamic Movement Primitives

In SE(3), dynamic movement primitives (Schaal, 2006; Ijspeert et al., 2013; Ude et al., 2014) represent robot trajectories as a second order dynamical system of the form:

$$\begin{array}{ll} \tau \left[\begin{array}{c} \dot{z}\\ \dot{\eta } \end{array}\right] & =\left[\begin{array}{c} \alpha_{y}\left(\beta_{y}\left(g-y\right)-z\right)\\ \alpha_{o}\left(\beta_{o}2\log\left(g_{o}*\bar{q}\right)-\eta \right) \end{array}\right]+\left[\begin{array}{c} f_{p}\left(x\right)\\ f_{o}\left(x\right) \end{array}\right] \end{array}$$ (20)

$$\begin{array}{ll} \tau \left[\begin{array}{c} \dot{y}\\ \dot{q} \end{array}\right] & =\left[\begin{array}{c} z\\ \frac{1}{2}\eta *q \end{array}\right] \end{array}$$ (21)

$$\begin{array}{ll} \tau \dot{x} & =-\alpha_{x}x, \end{array}$$ (22)

where $\alpha_{y},\alpha_{o}\in R^{+}$ and $\beta_{y},\beta_{o}\in R^{+}$ are constant gains, $g\in R^{3}$ and *g*_*o*_ ∈ SO(3) are attractor (goal) points for the positional and orientational components of the DMP, respectively, $\tau \in R^{+}$ is a time constant corresponding to the duration of the movement, *x* is a phase variable, *q* is a unit quaternion, and ∗ denotes the quaternion product. Notice that in the absence of terms *f*_*p*_(*x*) and *f*_*o*_(*x*), Eqs 20, 21 behave as a mass-spring-damper, and will converge towards *g* and *g*_*o*_ regardless of their initial condition. In this sense, constants *α*_*y*_, *α*_*o*_, *β*_*y*_ and *β*_*o*_ are usually chosen for critical damping. By adding the forcing terms *f*_*p*_(*x*) and *f*_*o*_(*x*), given by Eqs 23, 24, the system is modulated to fit an arbitrary trajectory, typically provided by demonstration. The forcing terms

$$\begin{array}{ll} f_{p}\left(x\right) & =\frac{\sum_{i=1}^{N}\psi_{i}\left(x\right)w_{i}^{p}}{\sum_{i=1}^{N}\psi_{i}\left(x\right)}x, \end{array}$$ (23)

$$\begin{array}{ll} f_{o}\left(x\right) & =\frac{\sum_{i=1}^{N}\psi_{i}\left(x\right)w_{i}^{o}}{\sum_{i=1}^{N}\psi_{i}\left(x\right)}x, \end{array}$$ (24)

$$\begin{array}{ll} \psi_{i}\left(x\right) & =\exp\left(-h_{i}{\left(x-c_{i}\right)}^{2}\right), \end{array}$$ (25)

consist of $N\in Z^{+}$ Gaussian radial basis functions (RBF) (Eq. 25) with centers *c*_*i*_, widths *h*_*i*_ and attached weight vectors $w_{i}\in R^{3}$ .

The canonical system Eq. 22, controls the time evolution of the system and at the same time guarantees convergence of Eqs 20, 21, as the influence of *f*_*p*_(*x*) and *f*_*o*_(*x*) will vanish as *x* → 0.

Given a demonstration trajectory $\left\{y_{\text{demo}},{\dot{y}}_{\text{demo}},{\ddot{y}}_{\text{demo}}\right\}$ , Eqs 20, 21 can be reformulated:

$$f_{p,\text{desired}}=\tau^{2}{\ddot{y}}_{\text{demo}}-\alpha_{y}\left(\beta_{y}\left(g-y_{\text{demo}}\right)-\tau {\dot{y}}_{\text{demo}}\right),$$ (26)

such that the open parameters in Eqs 23, 24, can be learned by, e.g., least-squares weighted linear regression. The same approach can similarly be applied to the orientation.

### 4.3 Encoding Task Dynamics

Process tasks, such as gluing, involve dynamic interaction with the environment and require that contact between the robot and the workpiece be maintained throughout the task execution. This implies that a target force and torque trajectory must be generated and inputted as a feed-forward signal into an appropriate robot controller (such as a hybrid or parallel position/force controller).

Typically, force-learning within the context of DMPs has consisted of running the robot task open-loop once the kinematic trajectory has been encoded, while the task interaction forces and torques are recorded. These force and torque profiles are assumed to correspond to the desired task profiles and can then be encoded as a mixture of RBFs (Nemec et al., 2013; Abu-Dakka et al., 2015).

As the kinematic trajectory taught by the user is not likely to be optimal, the forces and torques learned using the previously mentioned approach might not be desirable and therefore not result in the best possible task execution. This is particularly the case for process tasks such as gluing, where often a constant contact force is necessary in order to ensure even distribution of glue along the target surface. Thus, we propose to instead record the output of the surface normal estimator (see Section 4.1.1) during the user demonstration and encode this as RBFs:

$$n_{c,d}\left(x\right)=\frac{\sum_{i=1}^{K}\psi_{i}\left(x\right)w_{i}^{n_{c}}}{\sum_{i=1}^{K}\psi_{i}\left(x\right)}x.$$ (27)

The user can then manually input a desired force magnitude *F*_*in*_—which is often a known process variable—and this will then be applied in the direction of the surface normal, i.e. the desired force is *f*_*d*_(*x*) = *F* *_in_* *n*_*c*,*d*_(*x*).

### 4.4 Execution

Given the encodings of both the task kinematics (Section 4.2) and dynamics (Section 4.3) the controller chosen for task execution must ensure tracking of both the target position and orientation and the target forces and torques. Simultaneously, it must handle the prioritization of these two tasks in a way that guarantees both the successful outcome of the task and the stability of the system. Here, we apply segmentation in order to more effectively handle the different requirements of different stages of the task, as well as to allow us to apply strategies to increase the robustness of the execution.

The remainder of this section will present our approach to task segmentation and how it applies to task execution, and our proposed parallel position/force controller.

#### 4.4.1 Segmentation and Execution Flow

A key aspect to our overall approach is segmentation, whereby we divide the overall task into an *approach phase* and a *process phase* based on the output of the contact detector during the demonstration. The approach phase includes all robot motions prior to establishing contact with the target surface, while the process phase includes all in-contact interactions with the surface, as well as retraction motions where the robot leaves the surface. Technically, a third *retraction phase* could easily be defined to encompass these motions, but this has not been implemented in the current system iteration.

**FIGURE 4:**
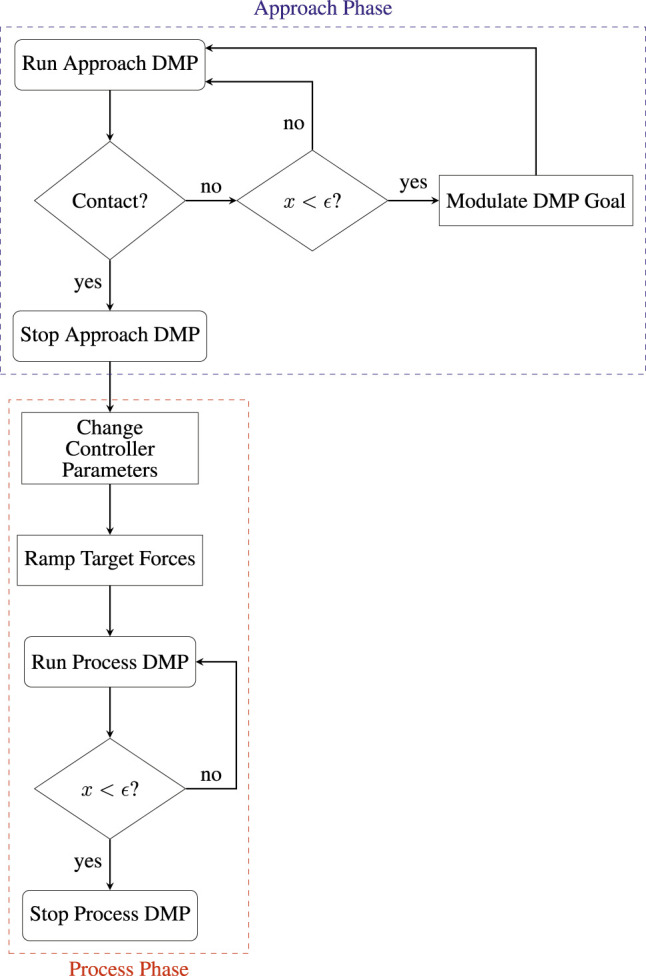
Block diagram of segmented execution flow.

Segmentation of the task phases is crucial to our method, as it allows us to use the semantic information contained in the different phases to apply appropriate control schemes and approaches to generalization. Namely, the execution is split into two separate DMPs, one for each of the phases, and these DMPs are run with different termination conditions. Figure 4 shows the segmented execution flow.

The approach DMP is modulated in a non-standard way by raising its goal a preset distance in the direction normal to the initial contact point in the surface. Once the robot reaches this point, if contact has not been established, the DMP goal is slowly moved down along the direction of the surface normal into the surface. This approach allows us to minimize impact forces, thus ensuring safe interaction with the PCB. A suitable trade-off between execution speed and impact forces can be achieved by regulating the speed of modulation of the goal, depending on the specific application. While this approach is similar to Conkey and Hermans (2019), note that they use it in a reactive manner, where the goal is modulated downwards towards the surface only in the case that contact is not established at the same point as it occurred in the demonstration, whereas we use it proactively in order to purposely reduce impact forces.

Once contact has been established with the surface, the starting point of the process DMP will be modulated to coincide with the robot position at contact, in this way avoiding discontinuities. Next, the parallel position/force controller parameters will be changed to appropriate values for the contact state. The target force will be obtained from the normal estimator, but instead of being directly inputted to the controller, will be ramped up slowly to avoid sudden overshoots. Finally, the process DMP will be run in parallel with the target forces obtained from the normal estimator. The next section will examine the details of the parallel position/force controller used for this purpose.

#### 4.4.2 Parallel Force/Position Control

**FIGURE 5:**
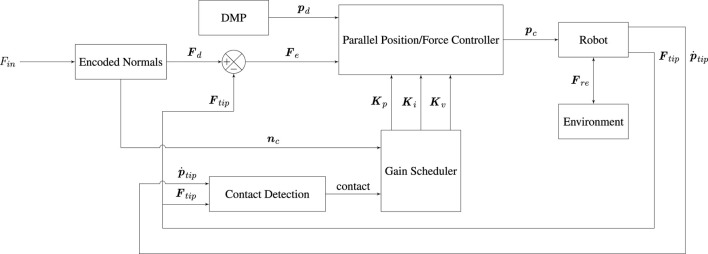
Block diagram of the controller architecture used for task execution.

The purpose of this section is to describe the parallel force/position control applied for the execution of the gluing behavior. The controller uses the standard formulation from (Siciliano et al., 2009, Section 9.4.3). The force-controller component is chosen to be a PIV control, i.e., a PI control on the force with velocity damping as explained in (Pérez-Ubeda et al., 2020). In addition, the controller gains are adapted according to the surface normal. The block diagram shown in Figure 5 shows the use of the controller during execution of the gluing task.

Notice the structural similarities with the admittance controller used for demonstration in Figure 3. The most notable differences are the removal of the normal estimation block and addition of a DMP and encoded normals block. The DMP provides the positional input to the parallel position/force controller. The encoded normals are used for two purposes: 1) In combination with an input process force, *F*_*in*_ provided by the user they specify the force input to the parallel position/force controller. 2) Through the gain scheduler, they specify the structure for the gain matrices of the parallel position/force controller, such that force-control is applied only in the axis perpendicular to the surface of contact, while the other two axes are purely position-controlled. Note that contact detection is also incorporated in a manner similar to during the demonstration. In this case, while contact has not yet been detected, the gains of the force-control component of the parallel position/force controller will be set to zero, such that the robot is purely position controlled. Once a contact is detected, gain-switching occurs, and the controller begins tracking the desired forces, *F*_*d*_, so as to minimize the force error, *F*_*e*_.

## 5 Experimental Evaluation

**FIGURE 6:**
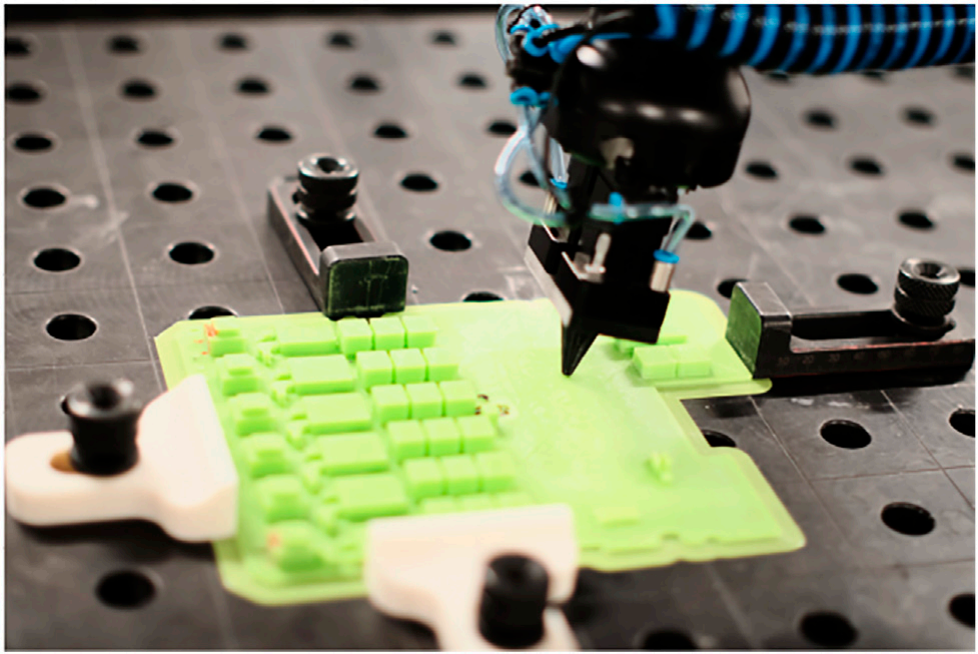
Robot executing the taught gluing-like task on our benchmark system. Notice the 3D-printed mock-up PCB (in green) and the 3D-printed mock-up gluing tip in a conical shape attached to the robot end-effector.

In order to evaluate the performance of our proposed system, we constructed a benchmark from a real-world use-case provided by a partner company. The original use-case involves gluing electronic components to a PCB. For our evaluation, we 3D-printed a replica of the PCB with the attached components. Similarly, we substituted the original gluing dispenser tip with a 3D-printed model. Both the PCB and gluing tip mock-ups can be observed in Figure 6. Note that, due to being 3D-printed, our mock-ups actually introduce more challenges from a control perspective than the original components. Printing imperfections on the surface of the PCB mock-up increase friction and introduce high frequency components in the forces that the parallel position/force controller must then address. The 3D-printed tip is also stiffer, which can challenge the stability of the system when compared to the more compliant true gluing tip.

We used a Universal Robots UR10e collaborative robot manipulator for our experiments. The robot is equipped with an internal force-torque (FT) sensor at the wrist 3 joint, close to the tool flange. All force-torque measurements and control were performed using the output of this sensor. The controllers were implemented in Python 3, using the *ur_rtde* ^1^ library for communication with the robot, both for streaming data and for sending commands to the internal robot controller. Our Python control application and the internal robot controller both run at a frequency of 500 Hz.

The following sections will evaluate our full system pipeline, from a demonstration of a task, as shown in Figure 1, to its segmentation, encoding and subsequent execution. We will present results for each of the system components previously described in Section 4. Note that all figures in the following sections are expressed in robot base frame, that is, with the *z*-axis normal to the table surface and its positive direction pointing out of the table, while the *x* − *y* plane coincides with the table plane.

### 5.1 Demonstration and Encoding

We will first evaluate the components of the system involved in the demonstration and task encoding pipeline. This consists of the surface normal estimator, damping adaptation scheme for the admittance controller, and DMPs for both the approach and process phases.

#### 5.1.1 Admittance Controller Parameter Adaptation

**FIGURE 7:**
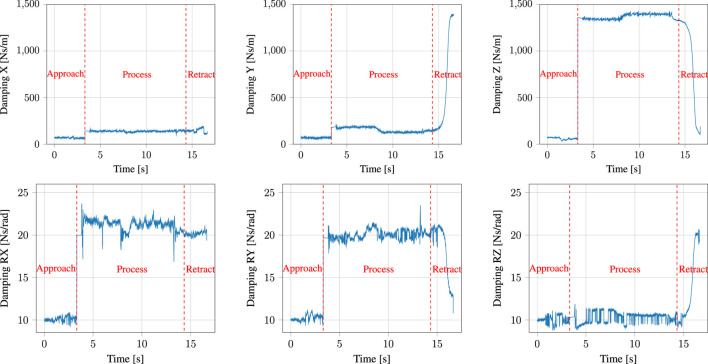
Damping adaptation throughout a demonstration of the gluing task.

Figure 7 shows adaptation of the damping term in the admittance controller used for kinesthetic teaching of the task, as outline in Section 4.1.2. Notice that the damping is varied slightly in all directions between *t* = 0 s and *t* ≈ 3 s, that is during the free air (approach) phase of the task. This is followed by an abrupt increase in damping in all axes of both the position and the orientation) at *t* ≈ 3 s, when contact with the surface of the PCB/table is detected. This ensures that the stability of the interaction is maintained despite the sudden increase in the environmental stiffness. The high damping value is maintained for another half a second, after which the parameters are allowed to vary again, albeit this time around the increased damping value as base value. This is particularly noticeable in the positional axes at *t* ≈ 8 s. Here, the user attempted to increase the speed of movement in a direction parallel to the plane of contact. This caused a decrease in damping in the *x*- and *y*-axes, accompanied by a slight increase in damping in the *z*-axis, as the robot was not moving in this plane. Once the robot leaves the surface during the retraction motion at *t* = 15 s and is moved again in free air, the damping in the *z*-axis very rapidly decreases. In all of these cases, the damping adaptation aims to assist the user in accomplishing their demonstration intention.

#### 5.1.2 Comparison of Admittance Controller vs. Freedrive for Demonstration

To test the usability of our adaptive admittance controller for demonstration, we devised an experiment where we compared it to the Free-drive control mode implemented in the Universal Robots. We programmed the robot to draw a reference figure-eight trajectory on a flat steel surface using a permanent marker. This was subsequently used as a visual reference in a small-scale user experiment consisting of six subjects. The subjects were instructed to kinesthetically teach the robot to follow the reference as closely as possible, without any requirements as to speed or time of the demonstration. Each subject repeated the demonstration three times with each controller, alternating between freedrive and our proposed adaptive admittance scheme.

To account for different timescales, we used Dynamic Time Warping (DTW) (Müller, 2007) to temporally align all demonstrations to the reference trajectory. We used DTW distance as a measure of deviation from the reference. Likewise, we measured the duration of the demonstration. The results are shown in Table 1, where each value is the average of the three attempts for that condition.

**TABLE 1:** Comparison of the mean distance from reference (lower is better) and mean duration (lower is better) between our adaptive admittance controller and Universal Robots’ freedrive controller for an experiment with six subjects. Each number is an average of three attempts performed by that subject.

	Mean distance from reference		Mean duration	
Subject	Adaptive admittance (ours)	Freedrive	Adaptive admittance (ours)	Freedrive
A	16.1	25.0	28.9	34.5
B	52.3	79.3	18.6	27.3
C	40.0	42.1	17.3	22.5
D	72.2	80.5	25.3	27.5
E	58.0	105.7	18.1	34.6
F	62.1	83.8	13.3	21.1
All	**50.1**	69.4	**20.2**	27.9

Although the sample size is not large enough to achieve statistical significance, the results show a clear trend that users achieve lower distances from the reference in a shorter amount of time when using our adaptive admittance controller compared to when using Freedrive.

#### 5.1.3 Surface Normal Estimator

**FIGURE 8:**
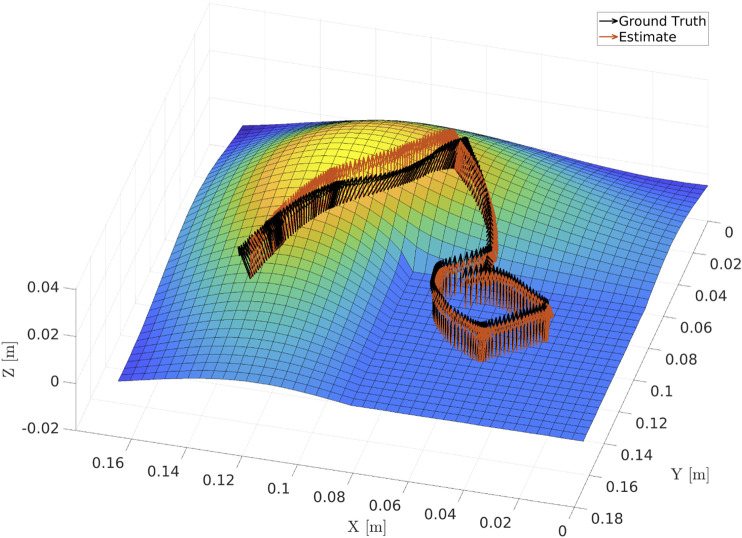
Output of the surface normal estimator compared to the ground truth value for a taught robot tool path along a curved surface defined by an eigenfunction of the L-shaped membrane.

**FIGURE 9:**
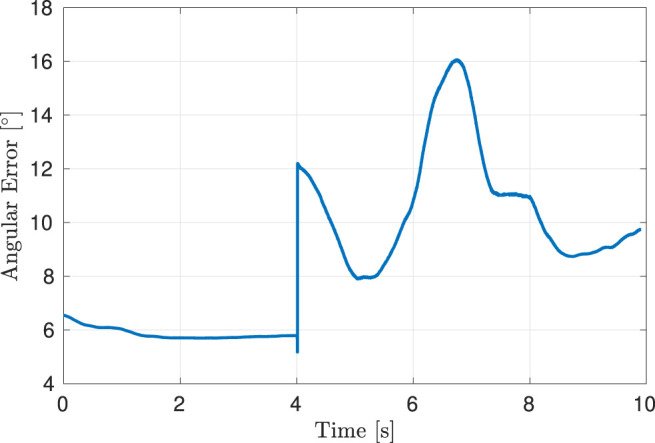
Estimation error for the surface normal compared to the ground truth.

We evaluated the surface normal estimator on a separate curved surface based on an eigenfunction of the L-shaped membrane, as shown in Figure 8. This surface is composed of a flat plane in one of its quadrants, which transitions into a smooth continuous function along the other three quadrants. Using a compliant controller, the robot was taught a motion along the surface, starting on the planar quadrant and moving onto and along the surface peak. The surface normal estimator was initialized to the value [0, 0, −1]. Figure 9 shows the evolution of the angular error along the path. An error of ∼6° is maintained along the planar quadrant of the surface. At *t* = 4 s the robot tool is moved onto the curved section of the surface, resulting in a discontinuous jump in the error up to ∼12° . This is to be expected, as the surface is in fact discontinuous at this point. The estimator then begins converging and the error decreases to 8° at *t* = 5 s. A maximum angular error of ∼16° is reached at *t* ≈ 6.5 s, coinciding with the area along the surface that exhibits the fastest rate of curvature change, after which the error decreases to ∼9° at *t* ≈ 8.5 s.

#### 5.1.4 DMP Encoding

**FIGURE 10:**
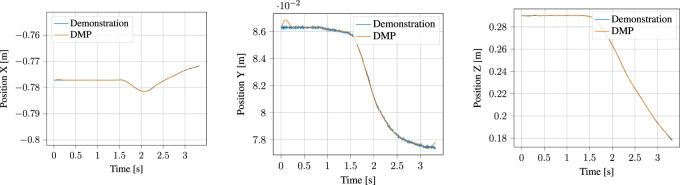
Demonstrated position and corresponding Position DMP for the approach motion.

**FIGURE 11:**
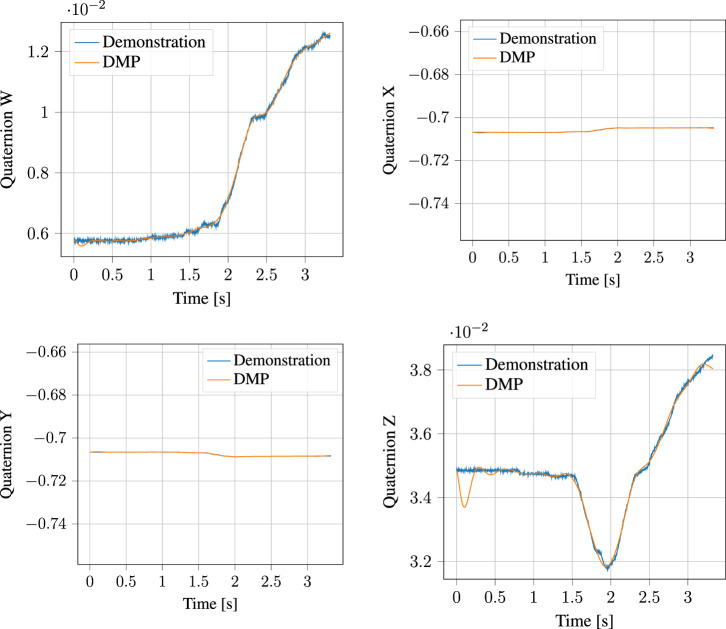
Demonstrated orientation (quaternion) and corresponding Quaternion DMP for the approach motion.

**FIGURE 12:**
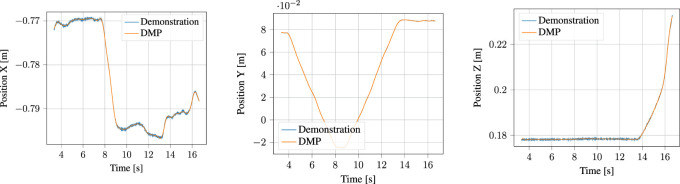
Demonstrated position and corresponding Position DMP for the process motion.

**FIGURE 13:**
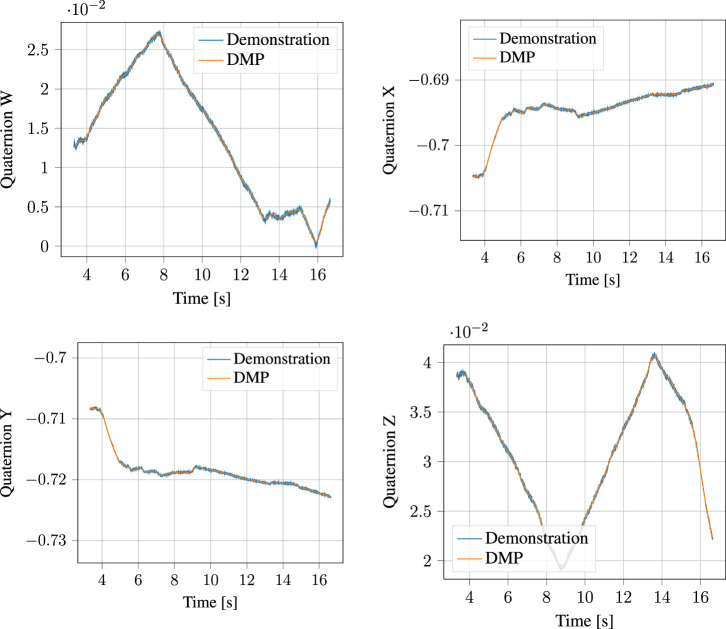
Demonstrated orientation (quaternion) and corresponding Quaternion DMP for the process motion.

The segmented approach phase of the demonstration, together with the output of the corresponding DMP learned from it are shown in Figure 10 for the position and Figure 11 for the orientation, respectively. Likewise, the process phase is shown in Figures 12, 13. Notice, particularly in the *y*-axis of component of Figure 10 and quaternion *w*- and *z*-components of Figure 11 that some error in the least-squares fit for the RBFs is present. However, at the scale this is apparent, it has little effect on the robot motion and is irrelevant to the approach phase overall.

### 5.2 Execution

Now that we have examined the components involved in demonstration and encoding processes, we will analyze the results of execution, particularly with regards to the performance of the parallel position/force controller.

**FIGURE 14:**
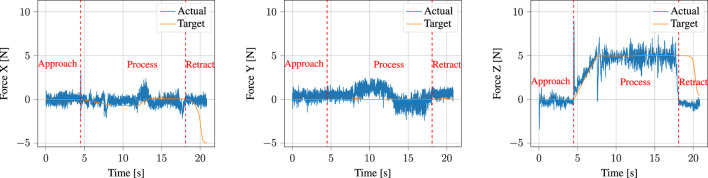
Target forces input to the parallel position/force controller (labelled *target*) compared to the actual measured forces (labelled *actual*). The dotted vertical red line shows the point at which contact with the surface was detected, upon which the gains of the parallel position/force controller are adapted and the forces are ramped to the target process force magnitude.

**FIGURE 15:**
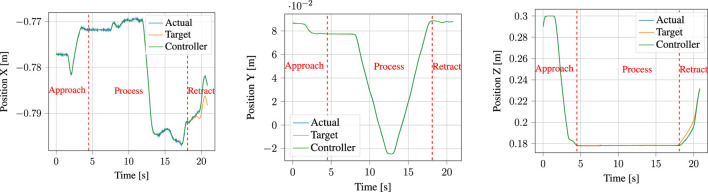
Target positions input to the parallel position/force controller (labelled *target*) compared to the total controller output consisting of combined position and force contributions (labelled *controller output*) and to the actual measured positions (labelled *actual*).

Figure 14 shows the target force reference of the controller, together with the actual forces measured by the robot f/t sensor end-effector during execution. The detected contact point with the surface of the PCB is indicated by a red dotted line at *t* ≈ 4.5 s. Note that the output of the normal estimator previously shown in Figure 8 multiplied by the user-inputted process force magnitude, which in this case was set to 5 N, is the target force. Furthermore, this means that the majority of the force control happens in the *z*-axis, as the robot was nearly perpendicular to the contact surface throughout the task. We will consequently focus most of our analysis on the *z*-axis component. During contact, the robot experiences an impact force of ∼9 N (together with ∼2.5 N in the *x*-axis). Such a low impact force is achievable due to our slow modulation of the approach DMP goal, as described in Section 4.4.1 and visualized in Figure 4. Following this, the force target is ramped up from 0 to 5 N between impact and *t* ≈ 7.5 s, after which it maintains a nearly constant process force of 5 N. Notice that after *t* ≈ 17.5 s, the robot is unable to maintain the target force. This is because the position reference moves it up and away from the contact surface, as can be seen in Figure 15. This can similarly be observed in the *x*-axis force, and is reflected again in the *x*-axis of Figure 15, as the prevalence of the force target means that the robot does not actually meet the position target. Since the retraction motion is not considered crucial to the successful completion of the task, it is currently not explicitly handled by the segmentation algorithm. Were it so, it would suffice with setting either the target force or the force control gains to zero in order to more precisely follow the position target.

A prevalent issue visible in Figure 14 is the amount of noise present in the FT sensor measurements. Notice that this is noticeable even before impact, at *t* < 4.5 s, when the robot is in free air. This shows that this is an issue in the sensor used in the Universal Robots themselves, and not in our system. Indeed, similar performance to our results here has been reported by, e.g. Pérez-Ubeda et al. (2020) when applying force control on Universal Robots arms.

## 6 Discussion and Perspectives

In the previous section, we evaluated our system on a benchmark inspired by a real industrial gluing use case, and analyzed the performance of the different system components both during a task demonstration and during the subsequent execution of the learned primitive. During demonstration, we showed that the adaptive damping in the admittance controller can assist the user in accomplishing their intended motion, while at the same time guaranteeing a stable interaction despite a changing environmental stiffness. Similarly, we showed that our surface normal estimator works satisfactory and allows the admittance controller to change the structure of its gain matrices, such that the normal direction maintains a higher damping to ensure stability, while the damping in the other directions is reduced to assist the user. During execution, we showed that our controller is able to track positional targets while applying the target force profile in the normal direction. We also showed the system’s ability to reduce impact forces by using DMP goal modulation.

However, there are many factors that were not fully considered and that could be improved upon in future iterations of the system.

When it comes to our adaptive admittance controller for demonstration, our small-scale usability evaluation in Section 5.1.2 shows a clear trend that our controller enables users to provide both more accurate and more efficient demonstrations of a path-constrained task when compared to Freedrive, as implemented on Universal Robots manipulators. This was verbally confirmed by the participants following the experiment. The subjects also reported that demonstrating was considerably more physically taxing using Freedrive, whereas our adaptive admittance scheme assisted in completing the task, making it easier to keep contact with the surface and to maintain a smooth continuous motion. As a full usability evaluation falls beyond the scope of this paper, backing this up with, e.g. the NASA-TLX (Hart and Staveland, 1988) or SUS (Brooke et al., 1996) tests is left for future work.

In gluing and dispensing tasks, it is often imperative for an optimal process outcome that the feed-rate be kept constant throughout the surface of application. While approaches that are able to adjust the feed-rate of the gluing gun to compensate for changes in robot speed exist ^2^, by far the majority of applications use constant feed-rate gluing guns and therefore require constant robot movement speed. With our approach, a skilled demonstrator can with some practice provide a near-constant speed demonstration, as is close to the case with the *y*-axis of Figure 15. However, it would be preferable to normalize execution speed in the encoding of the task to allow for imperfect demonstrations. Some approaches to DMP speed-scaling have been presented (Nemec et al., 2018a,b), typically through modulation of the time-constant, *τ*, with an additional term *ν*(*x*) that can be encoded as a mixture of RBFs. This method was considered for our system, but we were unable to achieve better performance than was demonstrated with it, as it did not result in constant speed. Alternative modulation schemes for DMP-speed will be investigated in future work.

The performance of the surface normal estimator is imperative for obtaining a good performance of the admittance controller used during the demonstration. It is of importance to both obtain fast convergence and little noise on the estimate. As seen in Figure 9, the convergence of the estimator might be improved; however, the parameters were chosen as a compromise between convergence rate and noise on the estimate. The surface normal estimation could potentially be improved by using a sliding model observer as presented in Hasan and Husain (2009). An analysis of the stability of the interconnection between the adaptive controllers and the surface normal estimator was omitted, as the faster dynamics of the controller when compared to those of the gain adaptation according to the surface normal estimator preclude instability. Note that the performance of the surface normal estimator still enables tracking of curved surfaces, as shown in Figure 8, despite its dynamics being slower than those of the controller.

In applications relying on force control it is important to ensure that force limits are not surpassed. Especially when relying on programming by demonstration there is a high chance that the nominal behavior will not comply with force constraints. Thus, it is relevant to modify the position/force controller such that it guarantees compliance with force constraints. This can e.g. be ensured by set invariance control as shown in Polverini et al. (2017). High forces will likely occur in the transition between free and constrained motions if small positional uncertainties are present. Therefore, it could be beneficial to adapt the reference path according to the measured force as proposed in De Wit and Brogliato (1997) with an adaptive approach. This would be an alternative approach to the goal modulation proposed in Section 4.4.1.

As our system relies heavily on the performance of several parameter estimators, particularly the surface normal estimator, a possible improvement would be to allow for repetitive update of our parameter estimates, for instance through the use of Iterative Learning Control (ILC) (Wang et al., 2009). Such methods have already shown good performance in learning from demonstration systems for peg-in-hole tasks (Nemec et al., 2013; Abu-Dakka et al., 2015).

## 7 Conclusion

In this paper, we have presented a full system for learning from demonstration of gluing and dispensing tasks that encompasses the full pipeline of demonstration, encoding and execution.

During the demonstration, our system is designed to assist the user and provide a non-obtrusive kinesthetic teaching experience. By adapting the damping of an admittance controller according to a force-velocity law, we aid the user during acceleration or deceleration. Furthermore, when contact against the target surface is detected, a surface normal estimation algorithm is used to adapt the gain matrices of the controller to guarantee higher damping and preserve stability in the normal direction, while allowing lower damping in the other directions. Unlike previous approaches in the literature, we do not assume diagonal gain matrices. Rather, we allow the whole eigenstructure to change. This results in lower user forces and better adaptation to the user’s intention.

During the encoding, we segment the task based on the initial contact into an approach phase and a process phase. Each of these are encoded as Dynamic Movement Primitives. Similarly, the surface normal estimate is encoded as Radial Basis Functions and synchronized with the process DMP phase system.

During the execution, a parallel position/force controller is used to meet both a position target and a force target, which is calculated based on a user-specified force magnitude applied on the encoded normal direction. The encoded normal is again used to vary the eigenstructure of the force controller gain matrices so as to apply force control in the contact direction and position control otherwise. Finally, we are able to reduce impact forces during the transition from approach to process by slowly modulating the goal of the approach DMP.

We have evaluated and analyzed our system on a benchmark based on a real industrial PCB gluing application and verified the suitability of our proposed methods, showing robust execution performance capable of meeting both the kinematic and dynamic requirements of the task. We have also shown that the surface normal estimator works satisfactorily on curved geometries, and that the adaptive controller used for demonstration results in faster and more accurate executions compared to a standard controller implemented on a collaborative robot.

## Data Availability

The raw data supporting the conclusion of this article will be made available by the authors, without undue reservation.

## Footnotes

1
https://gitlab.com/sdurobotics/ur_rtde.

2
https://aim-robotics.com/.
